# The utility of angiopoietin-2 and blood cell-derived biomarker indices in differentiating rapidly improving acute respiratory distress syndrome (RIARDS) phenotype from persistent-ARDS: a prospective observational study

**DOI:** 10.1080/07853890.2025.2596535

**Published:** 2025-12-08

**Authors:** Thejesh Srinivas, Vinutha R Bhat, Shwethapriya R, Pratibha Todur, Gagana Hanumaiah, Prithvishree Ravindra, Danavath Nagendra, Shobha U Kamath, Ashritha A Udupa, Shruthi Rao, Souvik Chaudhuri

**Affiliations:** ^a^ Department of Critical Care Medicine, Kasturba Medical College Manipal, Manipal Academy of Higher Education, Manipal, Karnataka, India; ^b^ Department of Biochemistry, Kasturba Medical College Manipal, Manipal Academy of Higher Education, Manipal, Karnataka, India; ^c^ Department of Respiratory Therapy, Manipal College of Health Professionals, Manipal Academy of Higher Education, Manipal, Karnataka, India; ^d^ Department of Obstetrics and Gynecology, Kasturba Medical College Manipal, Manipal Academy of Higher Education, Manipal, Karnataka, India; ^e^ Department of Emergency Medicine, Kasturba Medical College Manipal, Manipal Academy of Higher Education, Manipal, Karnataka, India

**Keywords:** Acute respiratory distress syndrome (ARDS;), rapidly improving acute respiratory distress syndrome (RIARDS;), angiopoietin-2, risk stratification, ICU survival

## Abstract

**Background:**

Rapidly improving acute respiratory distress syndrome (RIARDS) is defined as normalization of oxygenation (PaO_2_/FiO_2_ >300 mmHg) within 24 h of invasive mechanical ventilation. Angiopoietin-2 (Ang-2), a marker of endothelial dysfunction, may predict acute respiratory distress syndrome (ARDS) outcomes, but its role in RIARDS is unclear. This study evaluated Ang-2 and other biomarkers for predicting RIARDS and their association with survival.

**Research design and methods:**

In this prospective observational study (June 2023–January 2025) at a 2032-bedded tertiary centre, 193 consecutively enrolled mechanically ventilated ARDS patients were assessed. Demographic, clinical, and laboratory data were recorded on Day 1. Plasma Ang-2 was measured using a high-sensitivity ELISA. Primary outcome was RIARDS; secondary outcome was ICU survival.

**Results:**

RIARDS occurred in 18.6% of patients, highest in mild ARDS (52.9%) versus moderate (19.2%) and severe (10.4%). Multivariable regression identified Ang-2 as an independent predictor. A cut-off of 5896 pg/mL yielded an AUC of 0.731 (sensitivity 57.3%, specificity 88.9%). Patients with Ang-2 < 5896 pg/mL were more likely to develop RIARDS (86.1% vs. 13.9%, p-value < 0.001). Kaplan–Meier analysis showed significantly better ICU survival in RIARDS.

**Conclusions:**

Early Ang-2 measurement may help differentiate RIARDS from persistent ARDS, enabling prognostic enrichment and personalized management.

## Introduction

Rapidly improving acute respiratory distress syndrome (RIARDS) represents a phenotype of acute respiratory distress syndrome (ARDS) characterised by improvement in the oxygenation level as measured by the arterial blood partial pressure of oxygen to fraction of inspired oxygen (PaO_2_/FiO_2_) to >300 mmHg within 24 h of invasive mechanical ventilation (IMV). Careful characterization and early identification of RIARDS serve two crucial purposes. First, patients with RIARDS may often be managed with a less aggressive and limited adjunctive interventions, whereas those with persistent-ARDS typically require a more aggressive and multimodal treatment approach. Second, it may help refine patient selection in clinical trials by ensuring that study populations are more homogeneous in this heterogenous disease [1]. This is important because patients with RIARDS often recover quickly with supportive care alone and including them in interventional trials can dilute the observed treatment effect, making potentially beneficial therapies appear ineffective. Recognizing RIARDS early, therefore, has direct clinical relevance both for tailoring bedside management strategies and optimizing trial designs in ARDS.

RIARDS has been associated with the hypoinflammatory ARDS subphenotype, characterized by significantly lower levels of interleukin-8 (IL-8), IL-6, and plasminogen activator inhibitor-1 [1–6]. However, biomarkers such as IL-6 and IL-8 have several limitations compared with angiopoietin-2 (Ang-2). These include their short half-life, which increases the risk of ARDS misclassification, their burst-like kinetics with transient spikes followed by rapid declines depending on therapy, their unstable longitudinal behaviour over time, and their lack of specificity for ARDS [7–10]. In contrast, Ang-2 is a sensitive biomarker of endothelial dysfunction in ARDS, demonstrating superior diagnostic and prognostic performance compared with other endothelial markers and reliably reflecting vascular permeability and injury [11,12].

Literature emphasizes the need for studies exploring predictors of RIARDS, as data on this phenotype remain scarce [1].

The aim of this study was to evaluate the diagnostic utility of inflammatory biomarkers in predicting RIARDS. Specifically, we sought to determine the diagnostic utility of Ang-2 and blood cell–derived biomarker indices for identifying the RIARDS phenotype in ARDS cohort. In addition, we examined the association between RIARDS and mortality outcomes among patients with ARDS. The primary outcome of interest was the occurrence of RIARDS, while the secondary outcome was survival among patients who exhibited the RIARDS phenotype.

## Methodology

We conducted a prospective observational cohort study in a 2032-bedded tertiary care set-up between June 2023 and January 2025. A total of 193 mechanically ventilated patients with ARDS, defined according to the Berlin definition, were enrolled consecutively after obtaining written informed consent from their legally authorized representatives. As per the Berlin definition, ARDS was diagnosed based on acute onset (within one week of a known clinical insult), bilateral opacities on chest imaging, respiratory failure not fully explained by cardiac causes, and hypoxemia classified by PaO_2_/FiO_2_ ratio thresholds: mild ARDS (PaO_2_/FiO_2_ 300–201 mmHg), moderate ARDS (PaO_2_/FiO_2_ 200–101 mmHg), and severe ARDS (PaO_2_/FiO_2_ ≤100 mmHg), all measured with a minimum positive end-expiratory pressure of 5 cm H_2_O [13]. For the purposes of this study, patients were subsequently categorized into two groups: rapidly improving ARDS (RIARDS) and persistent ARDS. RIARDS was defined as a phenotype of ARDS characterised by improvement in oxygenation, demonstrated by an increase in PaO_2_/FiO_2_ to >300 mmHg within 24 h of IMV [1]. The study was reviewed and approved by the Institutional Ethics Committee (IEC1: 294/2022) and was prospectively registered with the Clinical Trials Registry-India (CTRI/2023/05/052937). This study was conducted in accordance with the Declaration of Helsinki, 1975 as revised in 2024, and the manuscript adheres to the Strengthening the Reporting of Observational Studies in Epidemiology (STROBE) standards (Supplementary Material 1).

Adult ARDS patients aged between 18 to 90 years on IMV were included in the study. Patients were enrolled on the day of ARDS onset, which was defined as the day on which a patient receiving IMV first met the Berlin definition criteria for ARDS.

Patients were excluded if they had received corticosteroids or disease-modifying anti-rheumatic drugs (DMARDs) prior to enrolment (as these agents could alter inflammatory mediator levels), if they had haematological malignancies that could confound leukocyte counts, or if they were managed exclusively with non-invasive ventilation (NIV). Patients with COVID-19-related ARDS were also excluded due to their distinct pathophysiology, clinical course, and treatment protocols, which differ markedly from those of non-COVID ARDS. A subset of data from this cohort was previously used for a secondary analysis with a different research objective, the results of which were published while the present study was ongoing [14]. The current analysis addresses a separate hypothesis with distinct outcomes of interest.

Data were collected prospectively on Day 1 and Day 2 following ARDS diagnosis. Day 1 was defined as the first 24 h from the time a patient fulfilled the Berlin criteria for ARDS, and Day 2 corresponded to the 24- to 48-h period after ARDS diagnosis. Demographic data, including age, gender, and aetiology of ARDS, were recorded. Clinical characteristics such as Charlson comorbidity index (CCI) score, acute physiology and chronic health evaluation-II (APACHE II) score, sequential organ failure assessment (SOFA) score, modified nutrition in the critically ill (mNUTRIC) score, and modified nutrition in the critically ill along with C-reactive protein (mNUTRIC-CRP) score were obtained at baseline. Biomarkers including C-reactive protein (CRP), procalcitonin, and haematological parameters such as white blood cell (WBC) count, blood urea nitrogen (BUN), systemic immune-inflammation index (SIII), systemic inflammation response index (SIRI), neutrophil-to-lymphocyte ratio (NLR), platelet-to-lymphocyte ratio (PLR), monocyte-to-lymphocyte ratio (MLR), CRP-to-albumin ratio (CAR), and BUN-to-creatinine ratio (BCR) were retrieved from the electronic medical records. Arterial blood gas analysis results, including lactate and PaO_2_/FiO_2_ ratio, were recorded as part of routine clinical care. For the purposes of this study, only Day 1 parameters were included in the statistical analyses, whereas Day 2 measurements were obtained to aid in clinical classification and trend assessment only and were not used in the current analyses.

Blood samples were collected from patients within 6 h of ARDS onset in EDTA-coated tubes. Samples were centrifuged at 5000 rpm for 15 min at room temperature, and the separated plasma was stored at −80 °C until analysis. Plasma Ang-2 concentrations were measured using a commercially available enzyme-linked immunosorbent assay (ELISA) kit (Diaclone SAS, France). The assay had a sensitivity of 25 pg/mL, with intra-assay and inter-assay coefficients of variation of 3.8% and 6.2%, respectively, indicating high precision and reproducibility. The kit demonstrated high specificity, with no significant cross-reactivity or interference between human Ang-2 and related analogues. The assay readings were measured at 450 nm using a microplate reader (Thermo Scientific, Multiskan FC).

### Statistical analyses

This study represents a predefined secondary objective of a prospectively enrolled cohort of ARDS patients (*N* = 193) originally recruited for a primary study on predictors of mortality. To determine whether the available sample provided adequate precision for the diagnostic performance of Ang-2 in discriminating RIARDS from persistent-ARDS, we estimated the standard error (SE) of the AUC using the Hanley & McNeil method [15]. Assuming an expected AUC of 0.75 and an anticipated RIARDS prevalence of 21% (n_1_ ≈ 41, n_0_ ≈ 152) reported in previous literatures [1,16], the variance of the estimated AUC was 0.002218, giving SE ≈ 0.047 and a 95% CI for the AUC of approximately 0.66–0.84. This interval was considered sufficiently narrow to provide a clinically meaningful estimate of Ang-2 discriminatory ability; therefore, the available sample (*N* = 193) was deemed adequate.

Hanley and McNeil formula is as follows:

$$\text{Variance of estimated AUC}=\;\frac{A(1‐A)\;+\;(n_{1}-1)(Q_{1}-A^{2})\;+\;(n_{0}-1)(Q_{2}-A^{2})}{n_{1}\times n_{0}}$$

• Where, A = expected AUC• n_1_ = expected number of RIARDS cases• n_0_ = expected number of persistent-ARDS cases •

$$•\;Q_{1}=\frac{A}{2-A}$$

•

$$•\;Q_{2}=\frac{2A^{2}}{1+A}$$

•

$$\text{SE}=\sqrt{\text{Variance of estimated AUC}}$$

• 95% CI = A ± (1.96 × SE)

All statistical analyses were performed using IBM SPSS Statistics, version 31.0.0.0 (117) software. Continuous variables were assessed for normality using the Shapiro–Wilk test. Normally distributed data are presented as mean ± standard deviation (SD) and compared using the Student’s *t*-test, whereas non-normally distributed data are expressed as median (IQR) and compared using the Mann–Whitney U-test. Categorical variables are reported as frequencies and percentages and were compared using the Chi-square test. Descriptive statistics were used to summarize demographic and baseline clinical characteristics of the study population. Multivariable logistic regression analysis was performed to identify independent predictors of RIARDS. The *p*-value ≤0.05 was considered statistically significant for all analyses. Receiver operating characteristic (ROC) curve and precision–recall curve were constructed using significant continuous variables from the multivariable logistic regression to determine diagnostic performance and calculate the AUC cut-off values for RIARDS prediction. Kaplan–Meier survival curves were generated to compare intensive care unit (ICU) survival between the RIARDS and persistent-ARDS groups, with statistical significance assessed using the log-rank (Mantel–Cox) test, and hazard ratio of mortality was calculated. Missing data were minimal and were handled using complete-case analysis without imputation.

## Results

A total of 193 patients with ARDS were included in the analysis. Except for age, all study variables demonstrated a non-parametric distribution. Categorical variables are reported as frequencies and percentages, while continuous variables are presented as mean ± SD for age and median (IQR) for CCI, SOFA, APACHE II, mNUTRIC, and mNUTRIC-CRP scores, along with other baseline characteristics (Table 1).

**Table 1.** Demographics and other characteristics of ARDS patients.

Variables			Mean ± SD/ Number (%) /Median (IQR)
Age in years			54.72 ± 15.99
Age in gender groups (in years)		Male	55 ± 1 6.17
		Female	54 ± 15.65
Gender		Male	138 (71.5%)
		Female	55 (28.5%)
Cause of ARDS		Pulmonary	112 (58%)
		Extra-pulmonary	81 (42%)
ARDS Category		Mild	17 (8.8%)
		Moderate	99 (51.3%)
		Severe	77 (39.9%)
Mortality			109 (56.5%)
Dialysis Required			63 (32.6%)
Prone Ventilation Required			44 (22.8%)
RIARDS (PaO_2_/FiO_2_ > 300 mmHg by 24 hours of IMV)			36 (18.7%)
Comorbidities:	Type-2 Diabetes mellitus		83 (43%)
	Hypertension		84 (43.5%)
	CKD		21 (10.9%)
	CLD		10 (5.2%)
	COPD		28 (14.5%)
	IHD		23 (11.9%)
	Rheumatoid arthritis		2 (1%)
	CVA		13 (6.7%)
CCI score			3 (1–5)
SOFA score			11 (8–15)
APACHE II score			26 (21–31)
mNUTRIC score			6 (4–7)
mNUTRIC-CRP score			7 (5–8)
WBC count (10^3^/µL)			12.4 (7.9–19.05)
BUN (mg/dL)			30.84 (21.02–49.76)
CRP (mg/L)			150.16 (71.7–255.01)
SIII			1779 (728.5–4365.5)
SIRI			7.97 (3.4–17.25)
NLR			12.3 (6.44–23.53)
PLR			195.74 (87.46–416.97)
MLR			0.73 (0.40–1.29)
CAR			0.057 (0.02–0.10)
BCR			19.64 (13.25–29.90)
Angiopoietin-2 (pg/mL)			5894 (3713.5-7010)
Lactate (mg/dL)			19 (12.45-33)
PaO_2_/FiO_2_ (mmHg)			117 (79–160)
*Procalcitonin (ng/mL)			4.36 (0.99–16.45)

The total cases (N) for this analysis was 193 (Male: 138; Female: 55); *Procalcitonin values were available for *N* = 151 patients. ARDS: Acute Respiratory Distress Syndrome; AFI: Acute Febrile Illness; RIARDS: Rapidly Improving Acute Respiratory Distress Syndrome.; PaO_2_/FiO_2_: Partial pressure of oxygen in arterial blood/fraction of inspired oxygen; CKD: Chronic Kidney Disease; CLD: Chronic Liver Disease; COPD: Chronic Pulmonary Disease; IHD: Ischemic Heart Disease; CVA: Cerebro Vascular Accident; CCI: Charlson Comorbidity Index; SOFA: Sequential Organ Failure Assessment; APACHE II: Acute Physiology and Chronic Health Evaluation; mNUTRIC: modified Nutrition Risk in the Critically Ill; mNUTRIC-CRP: modified Nutrition Risk in the Critically Ill- C-Reactive Protein; WBC: White Blood Cell; BUN: Blood Urea Nitrogen; CRP: C-Reactive Protein; SIII: Systemic Immune-Inflammation Index; SIRI: Systemic Inflammation Response Index; NLR: Neutrophil-to-Lymphocyte Ratio; PLR: Platelet-to-Lymphocyte Ratio; MLR: Monocyte-to-Lymphocyte Ratio; CAR: C-Reactive Protein-to-Albumin Ratio; BCR: Blood Urea Nitrogen-to-Creatinine Ratio; SD: Standard deviation; IQR: Interquartile range.

Comparisons between RIARDS and persistent-ARDS groups are summarized in Table 2. Age, Chronic Obstructive Pulmonary Disease (COPD), APACHE II score, mNUTRIC score, mNUTRIC-CRP score, and Ang-2 levels differed significantly between the two groups (Table 2).

**Table 2.** Differences in the variables among the RIARDS and persistent-ARDS patients.

Variable	RIARDS (*n* = 36) Mean ± SD / Number (%)/Median (IQR)	Persistent-ARDS (*n* = 157) Mean ± SD / Median (IQR)	*P*-value
Age in years	50 ± 18.05	55.8 ± 15.34	**0.05** ^#^
Type-2 Diabetes mellitus	13 (36.1%)	70 (44.6%)	0.354**
Hypertension	11 (30.6%)	73 (46.5%)	0.082**
CKD	4 (11.1%)	17 (10.8%)	0.961**
CLD	0 (0%)	10 (%)	0.120**
COPD	1 (2.8%)	27 (17.2%)	0.027**
IHD	6 (16.7%)	17 (10.8%)	0.329**
Rheumatoid arthritis	0 (0%)	2 (1.3%)	0.496**
CVA	1 (2.8%)	12 (7.6%)	0.293**
CCI score	1.5 (0–4)	3 (1–5)	0.158*
SOFA score	11 (6.25–13.75)	12 (9–15)	0.095*
APACHE II score	22.5 (17–26)	26 (22-32.5)	0.001*
mNUTRIC score	4 (3–6)	6 (4–7)	<0.001*
mNUTRIC-CRP score	6 (4–7)	7 (6–8)	0.004*
WBC count (10 [3]/µL)	10.5 (8.2–15.82)	12.9 (7.8-20)	0.163*
BUN (mg/dL)	30.13 (19.5–49.41)	31.7 (21.49–49.76)	0.809*
CRP (mg/L)	157.22 (70.82–303.74)	147.09 (71.70–254.17)	0.758*
SIII	1256 (288.75-4788)	1814 (748.50–4365.50)	0.341*
SIRI	6.60 (3.19–13.93)	8.64 (3.52–17.64)	0.417*
NLR	16.69 (5.18–19.8)	12.33 (6.72–24.74)	0.253*
PLR	183.85 (49.31–387.36)	212.28 (91.88–421.55)	0.437*
MLR	0.67 (0.492–1.305)	0.75 (0.395–1.29)	0.793*
CAR	0.059 (0.027–0.115)	0.057 (0.027–0.100)	0.574*
BCR	19.96 (12.63–23.91)	19.63 (13.49–30.82)	0.364*
Angiopoietin-2 (pg/mL)	4185.5 (3181.75–5805.50)	6589 (4132.5–7278.5)	<0.001*
Lactate (mg/dL)	16.8 (12-23.9)	20 (12.7–34.9)	0.204*
Procalcitonin (ng/mL)	3.7 (1.145–19.69)	4.63 (0.95–16.44)	0.936*

#Student’s t-test; **Chi-square test; *Mann-Whitney U test; All bolded P-values are statistically significant (P-value ≤ 0.05); ARDS: Acute Respiratory Distress Syndrome; RIARDS: Rapidly Improving Acute Respiratory Distress Syndrome.; CKD: Chronic Kidney Disease; CLD: Chronic Liver Disease; COPD: Chronic Obstructive Pulmonary Disease; IHD: Ischemic Heart Disease; CVA: Cerebro Vascular Accident; CCI: Charlson Comorbidity Index; SOFA: Sequential Organ Failure Assessment; APACHE II: Acute Physiology and Chronic Health Evaluation; mNUTRIC: modified Nutrition Risk in the Critically Ill; mNUTRIC-CRP: modified Nutrition Risk in the Critically Ill- C-Reactive Protein; WBC: White Blood Cell; BUN: Blood Urea Nitrogen; CRP: C-Reactive Protein; SIII: Systemic Immune-Inflammation Index; SIRI: Systemic Inflammation Response Index; NLR: Neutrophil-to-Lymphocyte Ratio; PLR: Platelet-to-Lymphocyte Ratio; MLR: Monocyte-to-Lymphocyte Ratio; CAR: C-Reactive Protein-to-Albumin Ratio; BCR: Blood Urea Nitrogen-to-Creatinine Ratio; SD: Standard deviation; IQR: Interquartile range.

Univariate and multivariable logistic regression analyses for predictors of RIARDS are presented in Table 3. Variables with a significant *p*-value in univariate analysis (APACHE II score, mNUTRIC-CRP score, and Ang-2) were included in the multivariable model. Ang-2 remained a statistically significant independent predictor of RIARDS (*p*-value:0.002, adjusted OR:1.00, 95% CI:0.999–1.000). Although mNUTRIC score was individually significant in univariate analysis, it was not included in the multivariable model to avoid multicollinearity, as mNUTRIC-CRP already incorporates these parameters. The multivariable logistic regression model demonstrated good calibration (Hosmer–Lemeshow *p*-value = 0.367) and achieved an overall classification accuracy of 81.3%, indicating that 81.3% of patients were correctly classified for the occurrence of RIARDS.

**Table 3.** Univariate and multivariable logistic regression analysis for prediction of RIARDS (*n* = 36).

Univariate Analysis				Multivariable Logistic Regression		
Variables	*p*-value	OR	95% CI	*p*-value	Adjusted OR	95% CI
Age in years	0.052	0.978	0.956–1.000	–	–	–
COPD	0.056	0.138	0.018–1.048	–	–	–
APACHE II score	**0.002**	0.925	0.881–0.971	0.382	0.969	0.903–1.040
mNUTRIC score	<0.001	0.699	0.569–0.857	**–**	**–**	**–**
mNUTRIC-CRP score	**0.003**	0.761	0.637–0.910	0.417	0.900	0.697–1.162
Angiopoietin-2 (pg/mL)	**<0.001**	1.00	0.999–1.000	**0.002**	1.000	0.999–1.000

All bolded *p*-values are statistically significant (*p*-value ≤ 0.05); RIARDS: Rapidly Improving Acute Respiratory Distress Syndrome; COPD: Chronic Obstructive Pulmonary Disease; APACHE II: Acute Physiology and Chronic Health Evaluation; mNUTRIC: modified Nutrition Risk in the Critically Ill; mNUTRIC-CRP: modified Nutrition Risk in the Critically Ill- C-Reactive Protein; OR: Odds Ratio; CI: Confidence Interval; Adjusted OR: Adjusted Odds Ratio.

Table 4 depicts the occurrence of RIARDS across ARDS severity categorises, as defined by the Berlin criteria. RIARDS distribution varied significantly with ARDS severity (*p*-value <0.001, Cramer’s *V* = 0.294), being highest among mild ARDS (52.9%) and progressively lower among moderate (19.2%) and severe ARDS (10.4%) patients.

**Table 4.** Distribution of RIARDS across ARDS severity (Berlin criteria) groups.

ARDS Severity (Berlin Criteria)	N	RIARDS (n, %)	Persistent-ARDS (n, %)	*p*-Value
Mild ARDS	17	9 (52.9%)	8 (47.1%)	<0.001**
Moderate ARDS	99	19 (19.2%)	80 (80.8%)	
Severe ARDS	77	8 (10.4%)	69 (89.6%)	

**Chi-square test; Cramer’s V: 0.294; All bolded *p*-values are statistically significant (*p*-value ≤ 0.05); ARDS: Acute Respiratory Distress Syndrome; RIARDS: Rapidly Improving Acute Respiratory Distress Syndrome.

**Figure 1.**
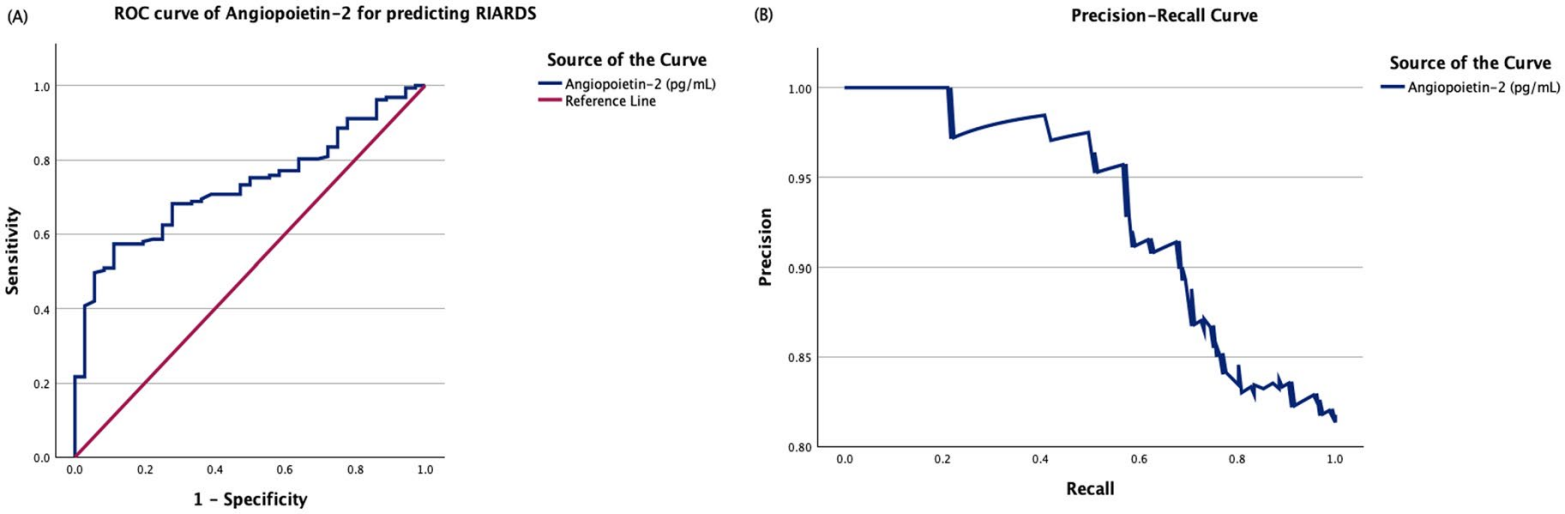
Diagnostic performance of Angiopoietin-2 for predicting RIARDS. (A) ROC curve demonstrating an AUC of 0.731 (95% CI: 0.656–0.806, *p*-value< 0.001) with an optimal cut-off of 5896 pg/mL (Youden’s Index = 0.462). (B) Precision–Recall curve showing precision of 88.8%, recall of 70.7%, and F1 score of 0.79 at a threshold of 4565 pg/mL. RIARDS: Rapidly Improving Acute Respiratory Distress Syndrome; ROC: Receiver Operating Characteristic; AUC: Area Under Curve

Diagnostic performance of Ang-2 for predicting RIARDS is represented in Figure 1. Figure 1(A) shows the ROC curve demonstrating an AUC of 0.731 (*p*-value< 0.001, 95% CI: 0.656–0.806, sensitivity: 57.3%, specificity:88.9%) with an optimal cut-off of 5896 pg/mL with Youden’s index of 0.462. Figure 1(B) depicts the Precision–Recall curve showing precision of 88.8%, recall of 70.7%, and F1 score of 0.79 at a threshold of 4565 pg/mL.

**Figure 2.**
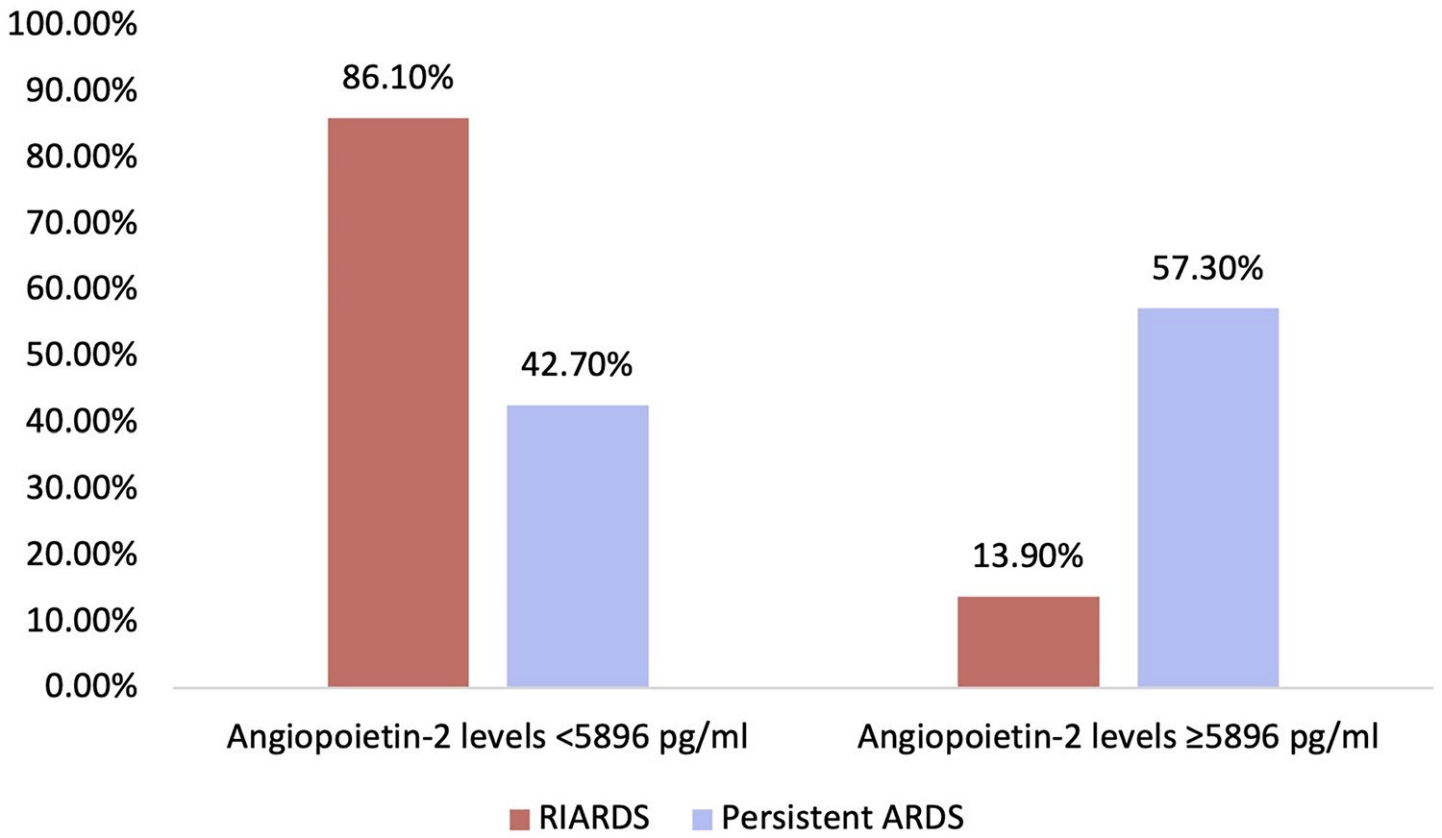
Association between RIARDS outcome and Angiopoietin-2 levels at 5896 pg/mL cut-off. Statistical test performed: Chi-square test; *p*-value < 0.001; RIARDS: Rapidly Improving Acute Respiratory Distress Syndrome.

Association analysis revealed a significant relationship between Ang-2 levels and RIARDS. Patients with Ang-2 levels < 5896 pg/mL progressed to RIARDS in 86.1% of cases, compared to only 13.9% among those with levels ≥ 5896 pg/mL (p-value < 0.001) (Figure 2). Univariate analysis further confirmed this association, showing that patients with Ang-2 levels ≥ 5896 pg/mL had 88% lower odds of progressing to RIARDS compared to those with Ang-2 levels < 5896 pg/mL (OR = 0.120, 95% CI: 0.044–0.325, p-value< 0.001), indicating that elevated Ang-2 is significantly associated with a lower likelihood of recovery from ARDS.

**Figure 3.**
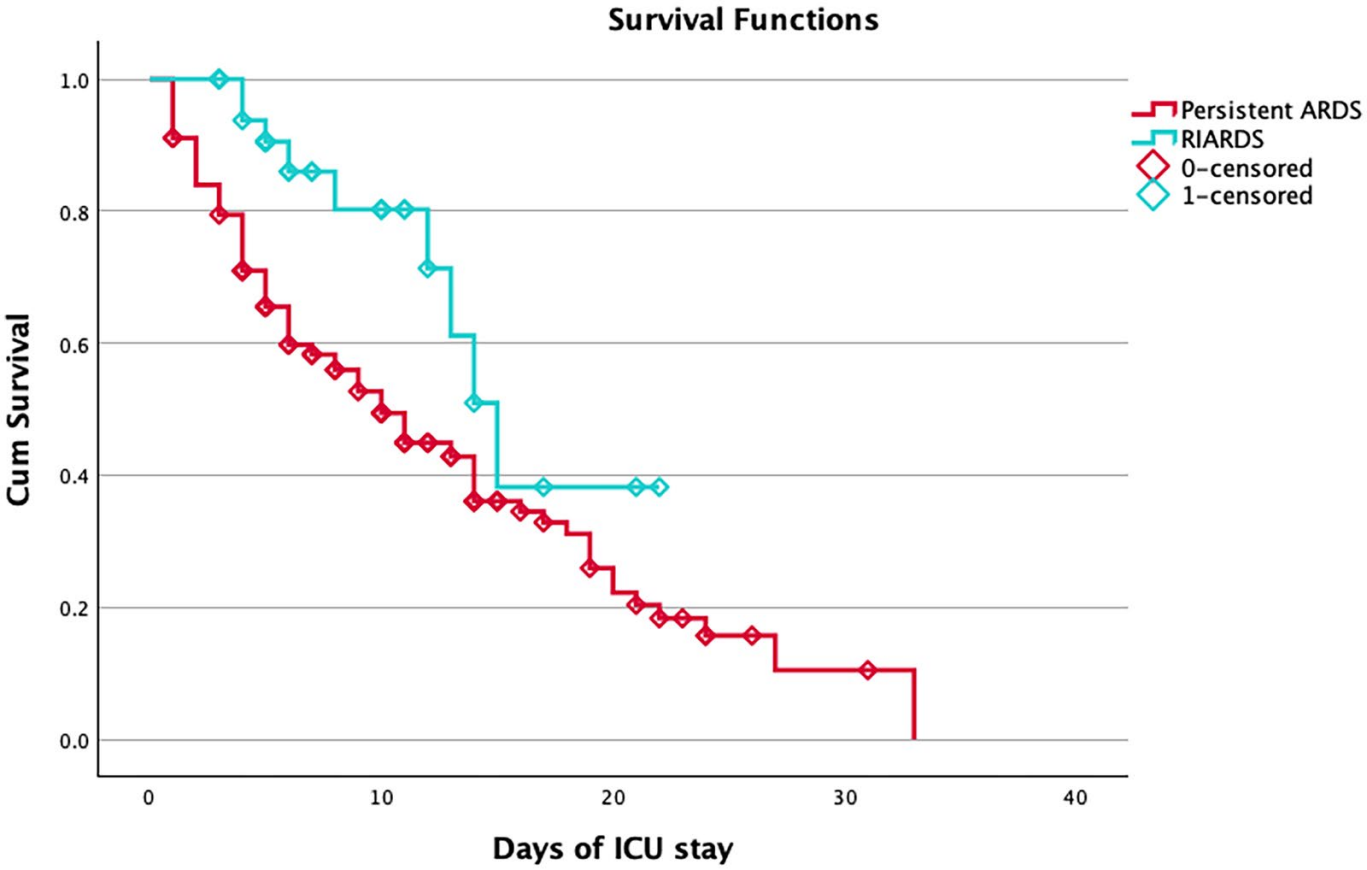
Kaplan–Meier survival curves comparing RIARDS and persistent-ARDS patients. RIARDS patients had significantly better survival (log-rank χ^2^ = 6.999, *p*-value: 0.008); RIARDS: Rapidly Improving Acute Respiratory Distress Syndrome; ARDS: Acute Respiratory Distress Syndrome; ICU: Intensive Care Unit

Kaplan–Meier survival analysis demonstrated a significant difference in ICU survival between RIARDS and persistent-ARDS groups (log-rank χ^2^ = 6.999, p-value: 0.008) as represented in Figure 3. The median survival time was 15 days (95% CI: 12.346–17.654) for RIARDS patients and 10 days (95% CI: 7.749–12.251) for persistent-ARDS patients. In Cox regression, RIARDS was associated with a significantly lower hazard of mortality (HR 0.42, 95% CI: 0.212–0.833, p-value= 0.013).

## Discussion

We observed that 36 out of 193 patients with ARDS (18.65%) had RIARDS, a finding comparable to the study by Valda Toro PL et al. who identified RIARDS in 21% of ARDS cases [1]. The underlying causes of RIARDS remain debated, ranging from non-inflammatory conditions such as atelectasis and cardiogenic pulmonary edema, which may lead to transient hypoxemia followed by rapid recovery, to its association with hypoinflammatory ARDS subphenotypes [1,2,17,18].

We found that Ang-2 levels ≥ 5896 pg/mL within 6 h of ARDS onset were predictive of persistent-ARDS, whereas lower levels (< 5896 pg/mL) were associated with RIARDS. Similarly, Valda Toro et al. reported significantly lower plasma Ang-2 levels in patients with RIARDS compared to those with persistent-ARDS [1].

Mechanistically, elevated Ang-2 levels aggravate alveolar oedema and inflammation by enhancing endothelial permeability and vascular leakage. By disrupting endothelial junction integrity, Ang-2 facilitates the flooding of protein-rich fluid into the alveolar space, a key process in ARDS-related oedema formation, while simultaneously impairing alveolar fluid clearance and thereby prolonging pulmonary oedema resolution [19–21]. Animal and human studies have demonstrated that elevated Ang-2 contributes to vascular leak and amplifies inflammatory responses [22]. The resulting persistent alveolar oedema and ongoing endothelial activation hinder inflammation resolution, making recovery slower and more difficult. These mechanisms may explain our findings that high Ang-2 levels were associated with persistent-ARDS, whereas lower Ang-2 levels were linked to RIARDS.

Elevated Ang-2 has consistently been the marker of several adverse outcomes in critically ill, especially in ARDS. Higher Ang-2 levels have been linked to prolonged ventilation, longer ICU or hospital stay, greater ARDS severity, and an increased risk of multiorgan dysfunction and mortality [9,10,19]. These associations highlight the potential value of Ang-2 as a prognostic biomarker capable of identifying patients at risk for a more complicated clinical course. Plasma Ang-2 is currently the most reproducible measurement, although bronchoalveolar lavage (BAL) Ang-2 has also been investigated, with further validation needed to determine its clinical utility [23]. Importantly, emerging therapies targeting the Ang-2/Tie-2 pathway highlights its translational relevance. A first-in-human trial of the anti-Ang-2 monoclonal antibody LY3127804 in patients with advanced solid tumours demonstrated acceptable safety and preliminary biological activity, supporting the feasibility of Ang-2–targeted therapeutics and suggesting potential applicability to ARDS populations in future studies [24].

We compared several blood cell–derived inflammatory biomarkers and indices, including NLR, PLR, SIII, SIRI, CAR, BCR, CRP, and procalcitonin, to evaluate their ability to predict RIARDS in resource-limited settings where Ang-2 measurement may not be feasible. However, none of these markers demonstrated predictive reliability comparable to Ang-2. This observation is consistent with evidence from the literature showing that early elevations in plasma Ang-2 are significantly more prognostic of subsequent ARDS development and 30-day mortality than nonspecific systemic inflammatory markers such as CRP, procalcitonin, WBC count, or the CAR [25]. In a large cohort study, Ang-2 levels measured within 24 h of hospital admission independently predicted ARDS onset after adjusting for baseline illness severity, highlighting its superior specificity for lung endothelial injury [9]. This observation aligns with findings from meta-analyses by van der Zee PA et al. indicating that while general inflammatory biomarkers (CRP, IL-8, RAGE, Von Willebrand factor) primarily reflect systemic immune activation, they lack the mechanistic association to vascular permeability abnormalities that Ang-2 directly represents [25].

The other significant finding from the present study was that RIARDS had a significantly higher prevalence in mild ARDS category (about 53%), as compared to moderate (19%) and severe ARDS category (10.4%). Notably, nearly one-third of RIARDS cases occurred in patients with moderate-to-severe ARDS, supporting the literature that not all mild ARDS cases represent RIARDS and not all moderate-to-severe ARDS cases necessarily progress to persistent-ARDS [18]. There are patients with moderate-to-severe ARDS who may experience rapid improvement in oxygenation (RIARDS) with timely and appropriate therapy, while at the other end of the spectrum, some patients with mild ARDS may fail to recover rapidly and thus can be classified as persistent ARDS. These observations underscore the importance of accurate phenotypic classification to guide management and prognostication.

We also observed that patients with persistent-ARDS had a higher hazard of mortality as compared to RIARDS, consistent with the findings by Schenck EJ et al. [18]. However, as the authors have concluded, the fact that persistent-ARDS has worse prognostic enrichment (outcome) may not mean that they have worse predictive enrichment (response to treatment) as well [18]. This distinction highlights the need for strategies that ensure patients with predictors of RIARDS receive timely, personalized therapy, while those with predictors of persistent-ARDS are identified early and offered aggressive therapy to maximize the potential for improvement.

Worsening organ dysfunction has been identified as a predictor of mortality in ARDS [26]. However, in this present study, although SOFA and APACHE II scores were significantly lower in the RIARDS group compared to the persistent-ARDS group, they did not independently predict RIARDS after adjustment in logistic regression analysis. A recent systematic review reported that hyperinflammatory ARDS carries a 2.5-fold increased risk of mortality compared with hypoinflammatory ARDS [27]. In contrast, we observed a substantially higher relative risk of mortality in persistent-ARDS (RR 5.26), suggesting that while elevated Ang-2 levels may help predict persistent-ARDS and lower levels may identify RIARDS, the underlying mechanisms are likely multifactorial and not solely attributable to the degree of systemic inflammation.

This present study had certain strengths. It is one of the few to prospectively evaluate RIARDS and examine the prognostic value of early Ang-2 levels. The systematic collection of clinical, demographic, and biomarker data at standardized time points, combined with multivariable logistic regression analyses to adjusted for confounders, enhances the validity and reliability of the findings, marking the strength of this study. Due to a class imbalance being present between RIARDS and persistent-ARDS, we plotted the precision-recall curve and calculated F1 score of Ang-2 to discriminate RIARDS and persistent-ARDS.

However, there were several limitations. The study was conducted at a single centre, which may limit external validity. Although the sample size was considered sufficient to estimate the discriminatory ability of Ang-2 with acceptable precision (95% CI of AUC ≈ 0.66–0.84), larger multicentre studies could further narrow the confidence interval and enhance generalizability. Additionally, Ang-2 levels were measured at a single time point in this study.

Future studies should explore serial Ang-2 measurements to better assess its predictive value and to understand the temporal dynamics of endothelial injury in ARDS. Performing repeated assessments and including additional endothelial biomarkers could offer further mechanistic insights. Future research should also focus on multicentre validation of Ang-2 cut-off values, and evaluating its use as a stratification biomarker in interventional trials may help identify patients who could benefit from therapies targeting endothelial stabilization. Investigating dynamic changes in Ang-2 throughout the course of ARDS could further clarify disease trajectory and therapeutic response.

## Conclusion

Early Ang-2 levels < 5896 pg/mL predicted RIARDS with good diagnostic accuracy, whereas higher levels were associated with persistent-ARDS. None of the commonly used inflammatory biomarkers or indices demonstrated comparable predictive performance. RIARDS was more frequent among patients with mild ARDS but was also observed in a subset of moderate-to-severe ARDS cases, underscoring the importance of accurate early classification. Persistent-ARDS was associated with significantly higher mortality, reinforcing its prognostic relevance. These findings highlight the potential utility of early Ang-2 measurement for risk stratification and personalized management of ARDS patients.

## Supplementary Material

Revised Supplementary Material 1 .pdf

## Data Availability

Anonymized data will be available from the corresponding authors upon reasonable request.
